# Is economic growth enough to propel rehabilitation expenditures? An empirical analysis of country panel data and policy implications

**DOI:** 10.1186/s12889-024-18601-y

**Published:** 2024-04-24

**Authors:** Rachel Neill, Hunied Kautsar, Antonio Trujillo

**Affiliations:** Johns Hopkins International Injury Research Unit, Health Systems Program, Department of International Health, Johns Hopkins Bloomberg School of Public Health, Johns Hopkins Bloomberg School of Public Health, 615 N. Wolfe Street Suite E8527, 21205 Baltimore, MD USA

**Keywords:** Rehabilitation, Health financing, Macroeconomics, Health policy

## Abstract

**Purpose:**

Rehabilitation is a set of services designed to increase functioning and improve wellbeing across the life course. Despite being a core part of Universal Health Coverage, rehabilitation services often receive limited public expenditure, especially in lower income countries. This leads to limited service availability and high out of pocket payments for populations in need of care. The purpose of this research was to assess the association between macroeconomic conditions and rehabilitation expenditures across low-, middle-, and high-income countries and to understand its implications for overall rehabilitation expenditure trajectory across countries.

**Materials and methods:**

We utilized a panel data set from the World Health Organization’s Global Health Expenditure Database comprising the total rehabilitation expenditure for 88 countries from 2016 to 2018. Basic macroeconomic and population data served as control variables. Multiple regression models were implemented to measure the relationship between macroeconomic conditions and rehabilitation expenditures. We used four different model specifications to check the robustness of our estimates: pooled data models (or naïve model) without control, pooled data models with controls (or expanded naïve model), fixed effect models with all controls, and lag models with all controls. Log-log specifications using fixed effects and lag-dependent variable models were deemed the most appropriate and controlled for time-invariant differences.

**Results:**

Our regression models indicate that, with a 1% increase in economic growth, rehabilitation expenditure would be associated with a 0.9% and 1.3% increase in expenditure. Given low baseline levels of existing rehabilitation expenditure, we anticipate that predicted increases in rehabilitation expenditure due to economic growth may be insufficient to meet the growing demand for rehabilitation services. Existing expenditures may also be vulnerable during periods of economic recession.

**Conclusion:**

This is the first known estimation of the association between rehabilitation expenditure and macroeconomic conditions. Our findings demonstrate that rehabilitation is sensitive to macroeconomic fluctuations and the path dependency of past expenditures. This would suggest the importance of increased financial prioritization of rehabilitation services and improved institutional strengthening to expand access to rehabilitation services for populations.

## Introduction

One in three persons could benefit from rehabilitation in their lifetime, but rehabilitative services continue to be underprioritized, particularly in low- and middle-income countries (LMICs) [1]. Rehabilitative care is rarely integrated into public financing mechanisms, suffers from limited budget allocation, and may be perceived as non-essential compared to other health care services [2]. In response, the World Health Organization’s Rehabilitation 2030 Call for Action emphasizes the importance of reducing unmet needs for rehabilitation services including the incorporation rehabilitation into Universal Health Coverage (UHC) initiatives and the expansion of public financing for rehabilitation services [3]. However, despite rehabilitation’s recent elevation on the global health agenda [4], inadequate resource allocation at the national level continues to be a challenge [5, 6].

How might countries increase their total expenditure for rehabilitation? Cross-country analysis indicates that economic growth and increased budgetary prioritization are the most common approaches to increase overall funds for health in LMICs [7]. Identifying the current influence of macroeconomic conditions is therefore important for understanding options for increasing rehabilitation financing.

Income elasticity assesses the relationship between the change in a country’s income and the demand for a specific good or service. It can be utilized to predict the relative importance of macroeconomic factors on rehabilitation expenditure. A 2020 analysis of the relationship between overall health care expenditure and gross domestic product (GDP) identified that approximately 43% of variation in overall health expenditure growth is attributable to economic growth. Income elasticity for health care services overall ranged from 0.65 in low-income countries, 0.88 in low-middle income, 0.93 in upper-middle income and 0.73 in upper-income countries [8]. Similar studies have produced elasticity estimates between 0.83 and 0.90 [9, 10]. This suggests that health care has a low elasticity (close to being inelastic); in other words, it is considered an essential good, particularly in low-income countries. Our paper’s primary contribution to the literature is addressing the absence of contemporary cross-country income elasticity estimates specifically for rehabilitative care. This fills a significant gap in the existing evidence, especially considering that rehabilitation services are frequently viewed as more of a luxury compared to other healthcare services [4]. This implies the possibility of a different income elasticity results for rehabilitative services specifically compared to the overall health expenditure estimates available in the literature.

In this paper, we aim to assess the relationship between macroeconomic conditions and rehabilitation expenditures to understand how much of the observed variation across countries is associated with GDP. We took advantage of having country panel data to control for time-invariant differences– heterogeneity across countries due to factors such as culture and geography. We determined the size and direction of income elasticity for rehabilitation services. We hypothesize that the income elasticity of rehabilitation services, while positive, is less than 1.

Improved understanding of these factors can support rehabilitation stakeholders to understand how sensitive rehabilitation expenditures are to macroeconomic fluctuations and how much economic growth or recession may impact the existing pool of resources for rehabilitative care.

## Materials and methods

Total current health expenditure and current health expenditure on rehabilitation were extracted from the World Health Organization’s (WHO) Global Health Expenditure Database (GHED), which provides internationally harmonized data on health expenditures. The variable for current rehabilitation expenditure data is an aggregate of three sources: domestic general government expenditures, external health expenditures, and domestic private health expenditures [11]. Rehabilitation expenditure is defined as the aggregate total for services for rehabilitation care, inpatient rehabilitation care, day care of rehabilitation, outpatient rehabilitation care, and services of rehabilitation home care [11]. The country panel dataset included expenditure data from 88 countries with reporting from the period 2016 to 2018, which was the longest period available.

For each country in the panel dataset, we obtained data on GDP (to represent macroeconomic conditions), population, a dummy for country income classification life expectancy, and the percentage of people above age 65 from World Bank Open Data [12]. As it is common in this literature, we used these variables together with total current health expenditure as controls. The description of all variables is provided in the World Bank database.

We applied multiple regression models to measure the relationship between macroeconomic conditions and rehabilitation expenditure. We checked the robustness of our income elasticity estimates by running four different model specifications: pooled data models (or naïve model) without control, pooled data models with controls (or expanded naïve model), fixed effect models with all controls, and lag models with all controls.

We considered the possible relevance of outliers in our analysis by running all models excluding countries with extreme values of GDP or rehabilitation expenditures. In all regressions, we compute robust standard errors to make inferences about the income elasticity parameter. Below, we will focus our discussion on the results for the most appropriate models– log-log specifications using fixed effects and lag-dependent variable models. In lay terms, log-log specifications use percentages to understand relationships while fixed effects accounts for unchanging differences across countries. The lag-dependent variable models consider the fact that changes in one variable might not immediately affect another. Overall, these tools help social scientists study complex connections like how economic growth affects rehabilitation expenditures over time. All estimations were performed using STATA software 17.

## Results

Table 1 identifies the size of the sample by country income classification and the mean per capita expenditure on rehabilitation by country income classification. This initial unadjusted analysis suggests a positive relationship when comparing middle-income and high-income countries, but a negative relationship when comparing low-income and middle-income countries.

**Table 1:** Average per capita expenditure on rehabilitation, by country income classification

Country income classifications	Average GDP per capita	Per capita rehabilitation expenditure^1^ Mean (St. dev.) Min-max	N
Low-income	$6,089.28	**6.63** (18.45) 0.00005–66.86	13
Lower-middle income	$6,974.96	**1.01** (1.88) 0.0001-7.29	23
Upper-middle income	$7,365.95	**5.51** (5.56) 0.004–17.51	19
High	$35,146.96	**115.64** (154.32) 0.24–492.90	33

*Notes*:

*Per capita expenditure has been averaged across the years 2016 to 2018, which were the years available for rehabilitation spending. N is the number of countries in each income category. We use three years of data for each country to compute mean and stdev*

**Fig. 1:**
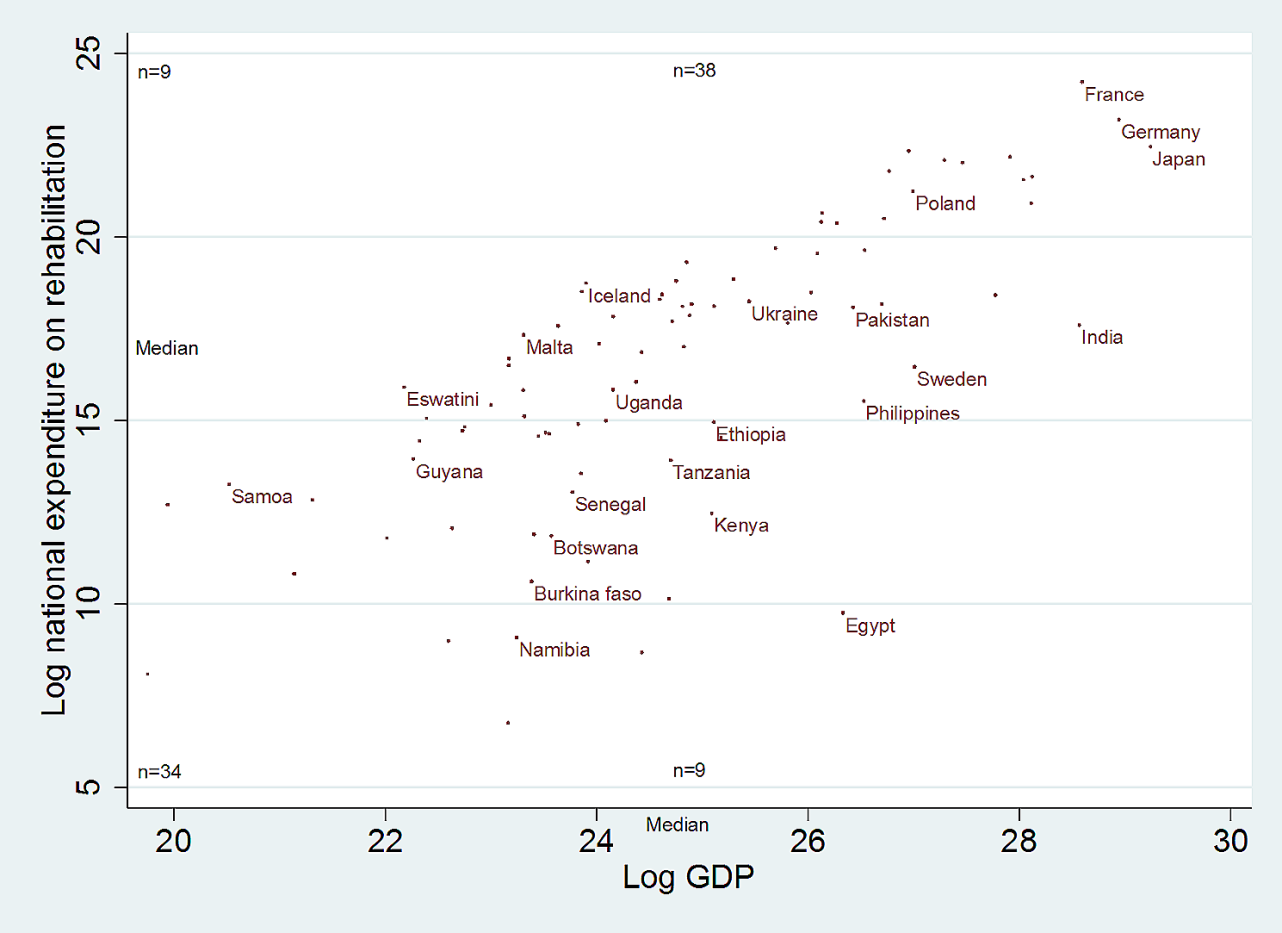
Association between GDP and national expenditure on rehabilitation (log scale; the size of the circle indicates population)

Taken in aggregate, the data indicates an overall positive relationship between the growth in log GDP and corresponding log national expenditure for rehabilitation (Fig. 1). Using the expanded naïve model, we find that approximately 75% of variation in expenditure can be explained by GDP. The results are similar when one uses other specifications.

Figure 1 also suggests that there are several countries (*n* = 9) that have allocated more resources than what their economy’s size would predict (e.g., Iceland, Malta). Equally interestingly, some countries (*n* = 9) have allocated less resources to rehabilitation than what their GDP would predict (e.g., Egypt, Kenya, Philippines).

Turning attention to the regression results, we conducted the Hausman test and reject the null hypothesis at *p* < 0.05 and proceeded to present the fixed effect results which partially controlled for endogeneity. Basically, rejecting the null in this test, implies that fixed effects model estimates are recommended since they will not be affected by omitted variable bias that appears in random effect models. We proceed to present the relevant estimates that control for omitted variable biased.

The estimates indicate a positive income elasticity for rehabilitation services, which remained significant once we control for country time-invariant unobservable factors. As expected, when one controls for the role of time-invariant differences within a country (e.g., culture, institutional settings, prevalence of war or humanitarian conflict and other time-invariant unobserved factors), the income elasticity estimates decline but remain positive. An additional effort to control for time-variant factors using the lag-dependent variable model also indicates a positive elasticity; yet, the parameter is still statistically significant and close to one. Taking together, the results from these models suggest that, in response to a 1% increase in economic growth, rehabilitation expenditure fluctuates between 0.9% as a lower bound and 1.362% as higher bound (Table 2). This suggests a moderate response, with our estimate close to and crossing the boundary of one.

**Table 2:** Income elasticity for rehabilitative services, by model specification (Log-log functions used in all models)

	Fixed-effects model	Lag-model
Income elasticity (Std. error)	0.90*** (0.345)	1.362*** (0.029)
R-squared	0.689	0.781
Sample size	211	176

*Notes*:

(***) p < 0.01; (**) *p* < 0.05; (*) *p < 0.10*

*All models include as controls total current health expenditure, population, country’s income classification data, life expectancy, percentage of people above age 65. In the lag-models, to control for time-variant unobservable factors, parameters are estimated using two waves of data resulting in a smaller sample size. All missing variables for the control variables were imputed at the mean value and a corresponding dummy for missing was added. Robust standard errors were computed*

It is important to highlight that our results quantify the increase in expenditures on rehabilitation that could be expected from countries’ differences– countries with higher economic growth have higher expenditures on rehabilitation. This does not imply that, within a country, rehabilitation expenditures will follow economic growth in the same proportions (even though we used country fixed effects) as some variations may be due to time-variant factors within the country. However, the within-country variation in rehabilitation expenditures from an economy’s growth will be close to the reported estimates.

This model can be used to forecast expected rehabilitation expenditure growth by projected GDP growth. For instance, according to our model, the International Monetary Fund’s (IMF) predicted annual economic growth of 5.9% in Uganda [13] may increase per-capita expenditures in rehabilitation to the range of 0.024–0.025 USD per capita. In Philippines, a similar economic growth rate will push per-capita expenditures to a range of 0.25–0.26 USD per capita. These projections highlight the likelihood that economic growth alone is likely not insufficient to move the sector out of its current trajectory of low overall expenditure due to low baseline levels of spending.

## Discussion

Our estimates identify an income elasticity around one for rehabilitative services. This suggests path-dependency for rehabilitation expenditures based on historical trends and some sensitivity to fluctuations in macroeconomic performance. Our estimate for rehabilitation's elasticity is higher than previous estimates for overall health care services [8—10] and divergent from the current global trend of health expenditure increasing at a faster rate than GDP [14].

A possible mechanism to explain the positive elasticity would suggest that lower economic growth reduces public funding into the rehabilitation sector. It is possible that the reduction in public budget reduces public expenditures while the private response in the provision of service is not sufficient to compensate the reduction in public expenditures. Under the assumption that private and public provision of services complement each other, a positive elasticity indicates that both sectors would shrink under a recession. Our findings therefore highlight outstanding questions on the dynamic of public and private response, which would be important for health policy makers to expand the provision of rehabilitation services in specific countries. This is a fruitful area for future research.

According to these estimates, we expect that economic growth across countries by itself is likely not sufficient to expand rehabilitation expenditures to cover population unmet needs in pursuit of UHC goals. This suggests that additional sectoral policy action and institutional strengthening will be necessary to accelerate the expansion of the sector and change the historical trajectory of rehabilitation expenditures to create a larger slope of growth.

Conversely, a recession across countries would imply a small percentage change in rehabilitation expenditures. However, because most countries in the panel dataset are starting from low baseline levels of expenditures, even minor expected decreases during a recession would need to be carefully managed to avoid future contractions in the sector.

### Strengths and limitations

A strength of our approach is that the WHO GHED provides harmonized, cross country, annual data which allowed us to use a panel dataset, improving overall sample size and precision.

A limitation of our analysis is the lack of available data on rehabilitation spending when compared to other health areas. The WHO GHED provides data from 192 countries from 2000 to 2019 []; however, our data set represents only 88 countries with available data from 2016 to 2018, which is a relatively short time horizon for the panel. The high R-squared from both the fixed-effects and the lag model indicates possible imputation within the rehabilitation expenditure data. For several countries, we do not have detailed information about the type of rehabilitation expenditures. For instance, recessions may contract inpatient care rehabilitation allocations more than outpatient rehabilitation services. This is an interesting area of exploration for future work. Finally, there is endogeneity between our variables which, although helped with the use of the panel dataset, is not overcome. Future research can expand on our associational findings through quasi-experimental methods to assess causal effects.

This study underscores the importance of investing in improved data systems to capture rehabilitative spending, as well as the broader importance of strengthening completeness of and capacities for national health accounts and other national expenditure tracking [15, 16]. In many countries, rehabilitation expenditures are additionally captured in non-health sector budgets, particularly social welfare agencies, and/or provided primarily through community-based rehabilitation programs. These realities further emphasize the importance of comprehensive approach to rehabilitation expenditure tracking. Future work can map the ‘sectoral location’ of rehabilitation across health and non-health Ministries, further improving the ability to make cross-country comparisons about rehabilitation expenditures. An understanding of the funding dynamics across health and social service agencies– whether complementary or substitutions– could also be further explored.

### Concluding remarks and policy implications

The primary conclusion of our study is that, according to our empirical findings, simply relying on economic growth in various countries is unlikely to be enough to enhance rehabilitation spending to a level that addresses the unmet needs of the population. How can rehabilitation stakeholders respond to these findings? Our model identified countries that are spending more (Iceland, Malta) or less (Egypt, Kenya) than predicted by their economy’s size. Future research could identify policy levers and health systems factors that contribute to the variation unexplained by our model. Rehabilitation advocates may consider targeting policy advocacy to expand the rehabilitation sector in countries where expenditures are at a lower level than predicted.

Our model also helps to predict the type of contraction that may be expected during a recession. This information could be relevant to policy makers in the rehabilitation sector to anticipate and smooth the effect of a recession in the rehabilitation sector.

Future work should also investigate the return of investment in rehabilitation, building on existing cost-effectiveness analysis [17], and can identify possible feedback loops between rehabilitation expenditures, improved labor productivity, and increased functioning that could further propel the economy’s growth.

In summary, this paper has argued that economic growth alone may not be sufficient to change the trajectory of rehabilitation expenditure in LMICs. Increasing coverage of rehabilitation services may require increased budgetary priority and efficiency gains to improve overall fiscal space, in tandem with implementing financing policies at the providers and consumer levels and strengthening institutional capacities.

## Data Availability

The data that support the findings of this study are openly available in World Health Organization’s (WHO) Global Health Expenditure Database (GHED) at https://apps.who.int/nha/database, and World Bank Open Data at https://data.worldbank.org.
